# Supplementary material for: Multi-stakeholder perspectives on reproductive and adolescent healthcare schemes in tribal regions of India: A qualitative study
Source: PLoS One. 2026 Feb 27;21(2):e0343794. doi: 10.1371/journal.pone.0343794 (PMC12948130; doi:10.1371/journal.pone.0343794)
Supplement: S2 Table — (DOCX) [file pone.0343794.s002.docx]

S2: Table, Codebook of the themes and sub themes developed

| **S.No.** | **Codes** | **Code Description** |
| --- | --- | --- |
| 1. | Absentism_HW | Apply this code for health worker absenteeism from the field/office. |
| 2. | Appropriate_Infrastructure | Apply this code for well-equipped, accessible, and properly maintained facilities essential for delivering effective healthcare services." |
| 3. | ASHA/AWW/ANM_Support | Apply this code for the support/assistance provided by community health workers. |
| 4. | Awareness_Required | Apply this code for the need of increasing knowledge and understanding among communities about health issues, available services, and preventive measures to improve health outcomes. |
| 5. | Awareness_RHS | Apply this code for increased awareness about Reproductive Health Services (RHS) among tribal communities. |
| 6. | Awareness_strategies | Apply this code for the different awareness strategies adopted in creating the awareness. |
| 7. | Cash_Incentives | Apply this code for the cash benefits received from the schemes. |
| 8. | Cultural_Barrier | Apply this code for the difference in beliefs, values, or practices that hinders effective communication or interaction between individuals from different cultural backgrounds. |
| 9. | Delayed_incentive_payments | Apply this code for the delay in the cash payments in form of incentives, from the government under the reproductive health schemes. |
| 10. | Existing_HealthSchemes | Apply this code for the existing health schemes refer to government or organizational programs currently in place to provide healthcare services and benefits to specific populations. |
| 11. | Family_support | Apply this code for the support from family members such as husband, inlaws, mother, father, and brother etc. to the adolescent girls and tribal women in the reproductive age group. |
| 12. | Frequent_Awareness | Apply this code for the frequent awareness sessions to the tribal population. |
| 13. | Gain_Trust_tribals | Apply this code for gaining the trust of tribal communities related to reproductive health scheme implementation. |
| 14. | Getting Awared | Apply this code for the tribal communities who are getting aware of the healthcare schemes. |
| 15. | Government_Support | Apply this code for the support from the government side to implement the reproductive healthcare schemes. |
| 16. | Healthcare_Workers | Apply this code for the existing health care workers/caregivers related to various mentioned schemes. |
| 17. | IEC_Customized_Tribalneeds | Apply this code for tailoring information, education, and communication materials to address the unique cultural, linguistic, and health needs of tribal communities. |
| 18. | Lack_Awareness | Apply this code for the lack of awareness related to the reproductive health schemes among the tribal communities. |
| 19. | Lack_Govt.support | Apply this code to refer the insufficient assistance, resources, or policies from the government to address specific needs or challenges. |
| 20. | Lack_GRS | Apply this code to refer the lack of grievance redressal system (complaint box). |
| 21. | Lack_Infrastructure | Apply this code to refers to the absence or inadequacy of essential facilities, systems, and services—such as healthcare centres, electricity, or communication networks—needed to support effective operations or quality of life. |
| 22. | Lack_Interest | Apply this code for the means not feeling curious, excited, or motivated about something. |
| 23. | Language_Barrier | Apply this code for the difficulty or inability to communicate effectively due to differences in language or dialect between individuals or groups. |
| 24. | Leader_Support | Apply this code for the guidance, encouragement, and resources provided by a leader to help individuals or teams achieve their goals or overcome challenges. |
| 25. | Maintaining_Records | Apply this code for the process of systematically organizing, storing, and updating important information or documents for future reference and use. |
| 26. | NGO_Collaborations | Apply this code for the collaboration from Non-Government Organisations. |
| 27. | Other_Problem | Apply this code for the other problems encountered among the tribal communities rather that reproductive health schemes. |
| 28. | Problem_Fund | Apply this code for the problem in having funds from the higher level. |
| 29. | Quality_Services | Apply this code to refer the provision of reliable, effective, and efficient services that meet or exceed the expectations and needs of the recipients. |
| 30. | School_awareness | Apply this code for the awareness reproductive health schemes in the schools. |
| 31. | Supervision | Apply this code for the act of overseeing, guiding, and managing the activities or performance to ensure tasks are completed correctly and efficiently in delivery of healthcare schemes. |
| 32. | Technology_Problem/HW | Technology problem/Health worker refers to challenges that health workers face in using technology, such as difficulties with digital tools, lack of training, or inadequate access to technological resources, which can impact their ability to deliver healthcare services effectively. |
| 33. | Traditional_Medicine/intervention | Apply this code to refer the healing practices, treatments, and remedies that are based on indigenous knowledge, cultural beliefs, and natural resources, often passed down through generations. |
| 34. | Training/orientation_IEC | Apply this code to refer the process of educating and preparing individuals, especially healthcare workers, on effective Information, Education, and Communication (IEC) strategies to promote awareness and behavioral change in target communities. |
| 35. | Transport_Problem | Apply this code to refer the problems related to the availability, accessibility, or reliability of transportation, which can hinder the movement of people or goods, especially in reaching healthcare facilities or remote areas. |
