# Supplementary material for: Multi-stakeholder perspectives on reproductive and adolescent healthcare schemes in tribal regions of India: A qualitative study
Source: PLoS One. 2026 Feb 27;21(2):e0343794. doi: 10.1371/journal.pone.0343794 (PMC12948130; doi:10.1371/journal.pone.0343794)
Supplement: S3 Table — (DOCX) [file pone.0343794.s003.docx]

S3: Table, Verbatim’s reported from the in-depth interviews

| **Themes** | **Codes** | **Verbatim’s** |
| --- | --- | --- |
| Challenges | Problem Fund | *"We use to maintain five registers, we have to buy on our own, no money they give for the register, from our 2000 Rs salary only we have to buy everything it is very difficult. Also, salary they don’t give on time, in two-three months they give. They used to say fund is not there."* |
|  | Lack of Government Support | *“Yeah, we used to maintain the records for everything, and I used to go and check all these things. But we used to buy register on our own, in Rs. 2000 what we all will do. At last we gives the bill but payment is very late,”* |
|  | Lack of Awareness | *“Also, only first-time pregnant lady will only get the incentives. There are many problems, they don’t have bank account or aadhar card also.”* |
|  | Language Barrier | *"Language problem is there, we know Hindi but tribal people have their language I am unable to understand sometimes what they are saying.”* |
|  | Transport Problem | *“Respondent: Ambulance if anyone calls at 108 will go and take them, all these are handled from Ranchi and Mamta Vahan is there but, in our block, the distance is so much between the villages and hospitals, so the ambulance don’t want to go everywhere”.* |
|  | Cultural Barrier | *"Some of them still prefer local food and medicines. There are some natural herbs we used to take. Because after taking native herbs we will not feel any discomfort."* |
|  | Absenteeism of Health Worker | *“We need more healthcare workers. ASHA and all they are not working, when they are in need or have to show report then only they come and talk to us."* |
|  | Lack of Infrastructure | *“We have problem with the infrastructure. We don’t have Anganwadi Centre building. See it is running in my home only. Higher officials are interested to construct it. But there is no land and space. No one is willing to donate their own land for Anganwadi building.”* |
|  | Technology problems for health workers | *“See one major problem these days are that everything is online, in my mobile, age 0-to-6-month child, and lactating mother child both are same, but we must fill data on two places. Where one place mother name is there, and another place child name is there. Hence from here many mothers don’t go there, so it very difficult to update both the places.* |
|  | Delayed incentive payments | *“Mother gets 1400 cash benefits after the delivery of child, and we gets Rs 2000 for registering the mother in the health centres, but payment for us is very late still we didn’t got payment of some deliveries”* |
|  | Lack of Grievance Redressal System | *"Interviewer: Okay, I want to know more about the complaint box. If you all want to complain anything regarding health, or facilities under health schemes where do you all usually go? Respondent: No, we never did anything like this before."* |
|  | Lack of Interest and mistrust on healthcare | *“I said earlier also people do not come in the awareness session, they says there is no place to sit where we will sit, we have our own work, we don’t want to waste our time in just listening the sessions. We are very much busy.”* |
|  | Other Problems | *“Respondent: Now, everything is available. Only thing if village people will restrict drinking hadiya (local alcohol), then everything will be fine. Husbands drinks more alcohol and it increases domestic violence more.”* |
| Collaborations | Local Leader Support | *but Yeah Mukhiya ji always comes and gives some awareness talks frequently on different schemes regarding health and other benefits.”* |
|  | Non-Governmental Organization (NGO) Collaboration | *"Yeah, Mukhiya ji and N plus foundation (Coded name) is working with us and help us when we are in need help towards health care."* |
| Health Accessibility | Existing healthcare schemes | *“Respondent: Now, facilities are there from government side related to schemes, such as iron tablets and all, also Kishori Yojana is running. Earlier yojana’s such as Yuva and Tejaswini yojana are also running.”* |
|  | Quality Service | *“Interviewer: Ok, I would like to know your opinion on the quality of the products given under the schemes?*  *Respondent: No, quality is very good. They never given the upcoming expiry medicines.”* |
|  | Technology problems for health workers | *“Interviewer: Ok, do you have any idea related to telemedicine or E Sanjeevni?*  *Respondent: Yeah, it was there in PHC here, but I think now it is not happening.”* |
|  | Traditional Medicines/Interventions | *“Respondent: There are some natural herbs we used to take. Because after taking native herbs we will not feel any discomfort. But after eating allopathic medicine we feel more weak, it will work immediately but strength will decrease. Although herbs take time but we don’t feel weak. Hence, we feels comfortable in taking natural herbs.”* |
|  | Transport Challenges in accessing healthcare services | *“Respondent: Ambulance if anyone calls at 108 will go and take them, all these are handled from Ranchi and Mamta Vahan is there but, in our block, the distance is so much between the villages and hospitals, so the ambulance wants to go everywhere.”* |
| Health Information | Awareness required | *“Government is doing good. But still tribal people are not aware of the incentives provided under the scheme.”* |
|  | Reproductive Health Scheme awareness | *“Could you please tell us about the different schemes for reproductive age group women?*  *Respondent: yes, we are there managing in health. Janani Suraksha Yojana, Mahila Samiti, a group meeting is there, another Anaemia Mukt Bharat. Earlier there was one yojana called Mata Samithi, now it became Jannai Suraksha yojana.”* |
|  | Awareness strategies | *"Yeah earlier we didn’t got, but now we didn’t got posters, related to yojanas. To us they didn’t give, to sahiya sathi they have given all these things. Now we use to do programs like saas bahu sammelan, every month and we use to get information about the nutrition and maternal health. In form of pictorial method, and through nukkad natak."* |
|  | Frequent awareness required | *"Under the absence of awareness about health and available healthcare schemes can result in poor health outcomes within the tribal women... Awareness will be generated, not at one time, two times three times, if again and again we will motivate them the awareness will generate."* |
|  | Reproductive Health awareness in schools | *“I got the incentives, but not that much. In school we used to get iron tablets, and sanitary pads in school for free. But I don’t feel that government is doing nice because we only got once to twice sanitary pad at college*.” |
|  | Lack of awareness towards reproductive health schemes | *“Respondent: yes, there are some schemes from that tribal population got benefitted, but still more awareness and trainings are required. Only thing is that full awareness is not there about the incentives among the village people especially among tribal population. Some schemes also giving cash benefits after childbirth, these all things are very good initiative but still people are not aware.”* |
| Health Service Delivery | Support from ASHA, ANM, & AWW | *"Some women know that we will give materials like nirodh, condoms, pads... but many women do not know the incentives, we have to go house to house and aware them."* |
|  | Support from government | *“Now facilities are there from the government side related to schemes, such as iron tablets and all, also Kishori Yojana is running.”* |
|  | Gain trust among tribals | *"See they are aware but some people are there still don't want to go, they tell that 'ok government will provide free ambulance for taking but after discharging from the hospital we have to come back on our own, who will bear the money, we don't have that much of money.'”* |
|  | IEC customized for tribal needs | *"I would suggest if you will do any awareness sessions, please provide some banners and posters with more pictures in it. It will help the tribal women to grab the content."* |
|  | Technology problems faced by healthcare workers | *"One major problem these days is that everything is online... it is very difficult to update both places.”* |
|  | Capacity building (Training & Orientation) | *“Interviewer: Did you all get training related to Schemes?*  *Respondent: Now, no training, but Yeah training has been given. In block, or in panchayat office it was given once in a year. Yeah, we used to get trainings once in a year, trainer used to come from district and train us about the schemes, through posters also.”* |
| Healthcare Workforce | Absenteeism in healthcare | *“Respondent: They are there in our village and working in awareness. We need more healthcare workers. ASHA and all they are not working, when they are in need or to show report then only they come and talk to us."* |
|  | Support from ASHA, ANM, & AWW | *"Yes, they visit once in a month, they also maintain registers and records of the pregnant and lactating mother too."* |
|  | Support from Government | *“Now facilities are there from the government side related to schemes, such as iron tablets and all, also Kishori yojana is running."* |
|  | Healthcare workers | *"See, now ANM is coming, ASHA is there, Sahaika (ASHA supporter) is there, I am there, Sahiya Sathi didi comes. So, it's manageable, no need for more healthcare workers.”* |
|  | Lack of Government support | *“Yes, we have given a letter, but no one has came from the higher level to look in this matter. We have place, and told to mukhiya also, he is telling from so long we will construct Aanganwadi, but no update.”* |
|  | Supervision/Inspection | *"Yeah, from CHC, doctors, block trainers, they use to come frequently to visit and check the record."* |
| Healthcare Financing | Cash Incentives | *"Yeah 1400 for JSY, they get and we also get incentives on time."* |
|  | Delayed cash incentives payments | *“Mother gets 1400 cash benefits after the delivery of child, and we gets rs 2000 for registering the mother in the health centres, but payment for us is very late still we didn’t got payment of some deliveries”* |
|  | Lack of support from government | *“See there is no Anganwadi centre, you are seeing, we are running Anganwadi in our home, but the problem is land, it is not available, and no one wants to donate their land for Anganwadi. This is the problem. The government is waiting for someone should donate the land then they will construct Anganwadi centre.”* |
|  | Problem fund | *"We don’t receive our cash incentives on time. Higher authorities say fund problem is there."* |
| Healthcare Infrastructure | Appropriate Infrastructure | *“We used to go to Anganwadi centre it is nearby. Infrastructure is nice. But there is no facility of fans. They gives medicines and edibles.”* |
|  | Lack of Infrastructure | *“Definitely, we have very poor infrastructure, see the building of Anganbadi centres, all are very old. In my panchayat I have total 5 Anganbadi only 1 Anganbadi have new building. Also there is issues of electricity and water supply which is also one of the hinderance in giving the facilities to the people in tribal areas.”* |
|  | Quality Service | *“Interviewer: Okay, in terms of quality of the product under the reproductive schemes, I want to know your opinion on the quality of the products?*  *Respondent: The quality of medicines was very nice. Also, those who do not take the medicines properly will not feel the benefit. For those who complete the medicine course of iron tablets, their Hb level will increase. Also, during delivery bleeding occurs so it's their problem if they do not eat medicine iron tablet they will suffer.”* |
|  | Health Technology Problem | *“See one major problem these days are that everything is online, in my mobile, age 0-to-6-month child, and lactating mother child both are same, but we must fill data on two places. Where one place mother name is there, and another place child name is there. Hence from here many mothers don’t go there, so it very difficult to update both the places.* |
| Leadership & Governance | Family Support | *“Now mother in-law are also aware of the balanced diet and supports their daughter in laws during their pregnancy."* |
|  | Support from Government | “*Respondent: Now government is constructing the healthcare buildings, earlier it was not there. XYZ foundation (Coded name) one was there (health centre) it near the road, in front of school.* |
|  | Lack of Government Support | *“Yes, we have given a letter to build Anganwadi centre, but no one has came from the higher level to look in this matter. We have place, and told to mukhiya also, he is telling from so long we will construct, but no update.”* |
|  | Support from local leader | *“Mukhiya ji and N plus foundation (coded name) is working with us and help us when we are in need help towards health care.”* |
|  | Supervision/Inspection | *"Sahiya Sathi and block trainer used to come frequently to check and inspect the record that we have maintained in the register."* |
