# Supplementary material for: Multi-stakeholder perspectives on reproductive and adolescent healthcare schemes in tribal regions of India: A qualitative study
Source: PLoS One. 2026 Feb 27;21(2):e0343794. doi: 10.1371/journal.pone.0343794 (PMC12948130; doi:10.1371/journal.pone.0343794)
Supplement: Attachment — Submitted filename: Reviewers response.docx [file pone.0343794.s005.docx]

**Response to reviewers:**

**Reviewer #1:**

The study is a well-planned qualitative study addressing a crucial region-specific lacuna. The leading questions and interviews have given answers to the questions raised. The study results can be noted and followed up by the stakeholders for addressing the lacunae.

Answer: We sincerely thank Reviewer 1 for their positive and encouraging feedback, and we appreciate the recognition of the study’s methodological rigor, region-specific relevance, and potential utility for stakeholders in addressing identified lacunae.

**Reviewer #2:**

1. Regarding the contribution of the study, the added value compared to previous studies in Indian populations is not clearly seen. It is suggested to include 1 or 2 paragraphs on which specific gap this study fills. This is in order for the reader to understand why the results matter for policies and programs.

- We thank the reviewer for this important suggestion. To clearly articulate the study’s added value, we have revised the *Introduction* sections by adding two dedicated paragraphs that explicitly highlight the specific gap addressed by this study.

2. Regarding the design, the analytical approach is not specified. It is suggested to state “qualitative descriptive design”, or “interpretive descriptive approach”, or “case study design”, or whatever was actually done. The use of a standard reference such as COREQ as a checklist should be added, and it should also be mentioned who designed the interview guides and how the pilot was carried out.

- We thank the reviewer for this constructive suggestion. We have now clearly specified the study as a qualitative descriptive design in the Materials and Methods section to enhance methodological clarity.
- Additionally, we have stated that the study reporting follows the Consolidated Criteria for Reporting Qualitative Research (COREQ) checklist, which has been added to the Methods section and included as a supplementary file.
- We have also clarified that the semi-structured interview guides were self-developed by the research team, informed by existing literature and study objectives, and that these guides were pilot tested among ten stakeholders to assess clarity, relevance, and cultural appropriateness before final data collection.

3. Regarding saturation, the process should be detailed, clarifying whether saturation was sought by group or in a general/global way. This is important to provide credibility to the study.

- We thank the reviewer for highlighting this important methodological aspect. We have now elaborated on the data saturation process in the Methods section, clarifying that saturation was assessed using a group-wise approach across stakeholder categories, as well as overall thematic saturation. Interviews were continued within each stakeholder group until no new codes or themes emerged, and cross-group comparisons were used to confirm saturation at the overall study level. These revisions have been incorporated to enhance transparency and methodological rigor.

4. Regarding the depth of the interviews, a duration of 10 to 15 minutes is reported. It is suggested to be more modest and use “semi-structured interviews” unless the guides were very focused and field notes were collected. Otherwise, this should be included as a limitation.

- We thank the reviewer for this important clarification. The study employed in-depth interviews using a focused interview guide; however, we acknowledge that the interview duration was relatively short (10–15 minutes) due to contextual field constraints. Many participants, particularly from tribal communities, provided brief or one-word responses, and several healthcare workers reported time constraints related to service delivery responsibilities, which limited prolonged engagement. We have now explicitly acknowledged this as a study limitation in the manuscript to ensure transparency and appropriate interpretation of the depth of findings.

5. Regarding the qualitative analysis procedure, it is not specified whether it was inductive, deductive, or mixed, nor the number of coders.

- We thank the reviewer for this important methodological clarification. We have now specified that the thematic analysis followed a mixed inductive-deductive approach. Initial coding was conducted inductively from the data, while deductive elements were guided by predefined health system domains relevant to reproductive and adolescent health schemes for category section. We have also clarified that multiple coders were involved in the analysis, with independent coding followed by team discussions to resolve discrepancies and refine the codebook. These details have been added to the Data analysis section to enhance transparency and rigor.

6. Regarding the discussion, it should be developed using relevant conceptual frameworks such as the health system, accessibility, etc. The same findings are repeated many times but with little theoretical depth.

- We have updated the discussion under the sub headings based on WHO building blocks of health system. To add the evidence we have tried to incorporate more depth with references to the discussion part.

7. In the ethics section, there are elements written in the future tense; the description should be unified in the past tense.

- Corrected.
